# Antimelanogenic Effects of Curcumin and Its Dimethoxy Derivatives: Mechanistic Investigation Using B16F10 Melanoma Cells and Zebrafish (*Danio rerio*) Embryos

**DOI:** 10.3390/foods12050926

**Published:** 2023-02-22

**Authors:** Hwang-Ju Jeon, Kyeongnam Kim, Chaeeun Kim, Sung-Eun Lee

**Affiliations:** 1 Red River Research Station, Louisiana State University Agricultural Center, Bossier City, LA 71112, USA; 2 Institute of Quality and Safety Evaluation of Agricultural Products, Kyungpook National University, Daegu 41566, Republic of Korea; 3 Department of Applied Biosciences, Kyungpook National University, Daegu 41566, Republic of Korea; 4 Department of Integrative Biology, Kyungpook National University, Daegu 41566, Republic of Korea

**Keywords:** curcumin derivative, melanogenesis, MC1R signaling pathway, zebrafish, melanin production, whitening agent

## Abstract

Regulation of melanin production via the MC1R signaling pathway is a protective mechanism of the skin of living organisms against exposure to ultraviolet rays. The discovery of human skin-whitening agents has been one of the most intense pursuits of the cosmetic industry. The MC1R signaling pathway is activated by its agonist, alpha-melanocyte stimulating hormone (α-MSH), and mainly regulates melanogenesis. Here, we evaluated the antimelanogenic activities of curcumin (CUR) and its two derivatives, dimethoxycurcumin (DMC) and bisdemethoxycurcumin (BDMC), in B16F10 mouse melanoma cells and zebrafish embryos. CUR and BDMC reduced the α-MSH-induced melanin production in B16F10 cells and also downregulated the expression of the melanin-production-related genes *Tyr*, *Mitf*, *Trp-1*, and *Trp-2*. Moreover, the biological activity of these two compounds against melanogenesis was confirmed in in vivo experiments using zebrafish embryos. However, the highest concentration of CUR (5 µM) resulted in slight malformations in zebrafish embryos, as indicated by acute toxicity tests. In contrast, DMC did not show any biological activity in vitro or in vivo. Conclusively, BDMC is a strong candidate as a skin-whitening agent.

## 1. Introduction

Natural products have been widely used in various fields, including biological, pharmaceutical, and nutraceutical areas, with the intention of obtaining and exploiting their beneficial effects, such as anticancer, anti-inflammatory, antifungal, antibacterial, and antimelanogenesis functions. These natural products are found in various organisms in Nature, including microorganisms, animals, and plants [1]. Plants are the main source of natural products, and as such the usage of plant extracts in the pharmaceutical and cosmetic industries has been increasing, partly owing to the negative perception associated with animal-origin extracts [1]. Numerous research groups are exploring the beneficial effects of new bioactive plant extracts or isolated plant-derived chemical compounds. Their studies attempt to demonstrate the biological mechanisms of valuable candidates, and suggest their potential usage. Investigating the underlying biological mechanisms of the active compounds of natural products is crucial for confirming the lack of harmful side effects in humans. In order to investigate the molecular mechanisms and potential toxicity of natural products, both in vitro and in vivo methods are employed, including cell cultures and animal experiments [1,2]. However, recently introduced ethics regarding the use of animals in research have enforced the pursuit of alternative animal models, such as small fish [2]. The zebrafish is an animal that adheres well to the requirements of alternative experimental animal models. Their genome is very similar to that of humans, they have a high reproduction rate, and their early development is achieved within 96 h after fertilization. Moreover, because of the clear chorion of the embryo, the entire developmental process can be easily observed [3,4,5]. As a result of these advantages, zebrafish have been used in various fields of biological research, including those of natural products.

Recently, the global cosmetic market has seen large growth [6]. In addition, whitening products are one of the most popular products in the cosmetics industry. Based on this, the exploration of new candidates for whitening agents is spotlighted in this field. Whitening agents play an important role in the cosmetic industry. Many studies have attempted to discover new agents, such as arbutin and kojic acid, with improved skin-whitening effects [6]. The main focus of such studies is to suppress the activation of the melanocyte-specific melanocortin-1 receptor-tyrosinase (MC1R-TYR) signaling pathway in melanocytes. The MC1R signaling pathway is the primary pathway regulating melanin production [7,8]. MC1R is activated by the alpha-melanocyte-stimulating hormone (α-MSH), adrenocorticotropic hormone (ACTH), and ACTH-secreting phaeochromocytoma (ASP) ligands [7]. Once activated, the α-MSH-induced cAMP pathway stimulates the activation of TYR [7,9]. The binding of α-MSH to MC1R on the cell membrane activates adenylate cyclase and leads to an increase in the levels of intracellular cAMP [7,9]. Subsequently, cAMP-dependent protein kinase A (PKA) phosphorylates cAMP response element-binding protein (CREB), leading in turn to the phosphorylation of MITF, which acts as a DNA-binding transcription factor of melanin-production-related genes, including TYR: a rate-limiting enzyme of melanogenesis. In particular, MITF regulates the expression of TYRP-1 and TYRP-2 proteins by binding to M-box in the tyrosinase distal element of these genes [7,10].

Curcumin (CUR), dimethoxycurcumin (DMC), and bisdemethoxycurcumin (BDMC) are active polyphenolic compounds of turmeric (*Curcuma longa*), a member of the ginger family, called curcuminoids. Although turmeric originates from India, it has been grown in many other places, including China and Southeast Asia [11,12]. Owing to its special taste and flavor, turmeric has been used over the past few centuries as a coloring agent, insect repellent, and antimicrobial agent. Because of the increased interest in natural products in the past several decades, turmeric has also been used as a nutraceutical, dietary supplement, and functional food [13,14]. Curcuminoids have been well known to exhibit a broad spectrum of bioactivities, including antioxidant, anti-inflammatory, antimicrobial, antifungal, and anticancer effects [14,15,16]. These beneficial effects have been established both in vitro and in vivo by many studies using cell lines and mouse models, respectively [11,13].

In this study, we evaluated the antimelanogenic effects of curcuminoids in vitro and in vivo using a mouse melanoma cell line (B16F10) and zebrafish embryo model, respectively, to explore new whitening agent candidates for use in cosmetics. Concomitantly, we performed acute toxicity tests to ensure the safety of using these compounds in vertebrates.

## 2. Materials and Methods

### 2.1. Chemicals and Reagents

CUR, DMC, BDMC, α-melanocyte stimulating hormone (α-MSH), and kojic acid were purchased from Sigma-Aldrich (St. Louis, MO, USA). All other reagents used in this study were of molecular biology grade.

### 2.2. Cell Culture

The B16F10 mouse melanocarcinoma cell line was purchased from the American Type Culture Collection (ATCC, Manassas, VA, USA). Culturing cells were performed as described before [17]. Briefly, cells were cultured in Dulbecco’s modified Eagle’s medium (DMEM, GE Healthcare, Chicago, IL, USA) containing 10% fetal bovine serum (Corning, Corning, NY, USA) and 1 % penicillin/streptomycin (GE Healthcare, Chicago, IL, USA). Cells were cultured in a CO_2_ incubator (BINDER, Tuttlingen, Germany) under conditions of humidified atmosphere with 5% CO_2_ at 37 °C. Cells used in experiments were kept at as low a passage number as possible.

### 2.3. Cell Viability Assay

The proliferation of B16F10 cells was evaluated using the CellTiter 96 Aqueous One Solution Cell Proliferation Assay Kit (Promega, Madison, WI, USA) according to the manufacturer’s instructions [17]. Specifically, 1 × 10^3^ cells were seeded on a 96-well plate and cultured for 24 h to recover. After recovery, the medium was changed with fresh medium containing CUR (5, 10, 20, 40, and 80 μM), DMC (5, 10, 20, 40, and 80 μM), or BDMC (5, 10, 20, 40, and 80 μM). Treated cells were incubated for an additional 48 h and then 20 μL of MTS solution per 100 μL media was added to each well. After an additional incubation for 4 h, the plate was placed at 25 °C for 30 min, followed by determining absorbance at 490 nm using the Multiskan GO microplate spectrophotometer (Thermo Fisher Scientific, Waltham, MA, USA). Data were normalized to the absorbance value of the control and represented as percentage ratio.

### 2.4. Determination of Melanin Contents

Total melanin contents were determined according to the method previously reported [18]. Briefly, B16F10 cells were pretreated with 200 nM α-MSH to induce melanogenesis, and then incubated for 72 h with phenol-red-free DMEM supplemented with 10% FBS and antibiotics. Kojic acid (200 μM), CUR (5 and 10 μM), DMC (5 and 10 μM), or BDMC (5 and 10 μM) was added to the media. After incubation, media were collected for measuring extracellular melanin contents, whereas 200 μL RIPA lysis buffer was added to harvested cells for determination of intracellular melanin contents. To isolate intracellular melanin, lysed cells were centrifuged at 4 °C and 13,000× *g* for 15 min and the pellet was resolved in 10% DMSO solution containing 1 N NaOH at 95 °C for 2 h. Melanin contents were determined by measuring absorbance at 470 nm using a spectrophotometer. For normalization of samples, protein concentration was determined by the BCA method as previously described [18].

### 2.5. RNA Isolation and qRT-PCR

Isolation of total RNA and determination of the level of mRNA was performed using the QuantStudio 3 Real-Time PCR System (Applied Biosystems, Foster City, CA, USA) according to a previously described method [19]. Briefly, total RNA was extracted from samples using the Trizol reagent (Ambion, Austin, TX, USA), and the absorbance at 260 nm, 260/280 nm, and 260/230 nm was measured for quantity and quality check of extracted RNA using the Multiskan GO microplate reader. Subsequently, 5 μg cDNA was synthesized from extracted total RNA using the Maxima first strand cDNA synthesis kit (Thermo Fisher Scientific, Waltham, MA, USA). The obtained cDNA was used as the template for the determination of the levels of *Mitf*, *Tyr*, *Trp-1*, and *Trp-2* mRNAs. Accordingly, qRT-PCR was performed using the Luna Universal qPCR Master Mix (New England Biolabs, Ipswich, MA, USA), according to the manufacturer’s instructions. The primers used in qRT-PCR are listed in Table S1. All data were normalized to the level of *Gapdh* mRNA.

### 2.6. Intracellular Tyrosinase Activity Assay

The activity of intracellular tyrosinase was determined according to a previously reported method [18]. Briefly, α-MSH-pretreated B16F10 cells were incubated with various concentrations of test compounds (200 nM kojic acid; 5 and 10 μM CUR, DCM, or BDCM) for 72 h. Cells were then harvested in a lysis buffer containing proteinase inhibitors and centrifuged at 13,000× *g* and 4 °C for 15 min to collect the supernatant. For measuring the oxidation rate of L-DOPA, 20 μL of collected sample and 80 μL of 40 mM L-DOPA were mixed and placed in each well of a 96-well plate. Samples were incubated at 37 °C for 2 h and their absorbance was measured at 475 nm using a microplate reader. Absorbance values were normalized to the protein concentration of each sample.

### 2.7. Zebrafish Embryo Test

AB strain zebrafish were generously provided by Prof. Tae-Lin Huh from the Korean Zebrafish Resource Bank, Kyungpook National University (Daegu, Republic of Korea) and kept in the laboratory in an automatic flow-through system under conditions of 26 ± 1 °C and 8 h:16 h day and night ratio for more than eight generations. Fish were cared for according to the modified ZFIN general fish care method previously reported [19]. The night before the experiment, 10 pairs of fish were mated in order to obtain embryos. Early in the morning, embryos were collected, and healthy embryos (>80% fertilization ratio) were transferred to E3 media. Fifteen healthy embryos were placed in each well of a 6-well plate, followed by the addition at the shield stage of E3 media containing kojic acid (8 mM) and test compounds (CUR, DMC, or BDMC; 1.25, 2.5, and 5 μM each). After treatment, the developmental stage, abnormal development, and phenotype were checked every 24 h until 72 hpf. The phenotype of embryos was photographed at 72 hpf using an Olympus BX53 microscope equipped with a DP80 CCD camera (Olympus, Waltham, MA, USA). After documentation, embryos were homogenized with CETi lysis buffer (Translab, Daejeon, Republic of Korea), and melanin concentration was determined by measuring absorbance at 475 nm using a microplate reader.

### 2.8. Statistical Analysis

All statistical analyses were performed using GraphPad Prism 8.0 (GraphPad Software, San Diego, CA, USA). One-way ANOVA with Tukey’s post hoc test was performed for multiple comparisons. All data are presented as the mean ± standard deviation (SD). A *p* < 0.05 was considered statistically significant. 

## 3. Results

### 3.1. Cytotoxic Effect of Curcumin Derivatives in B16F10 Cells

**Figure 1:**
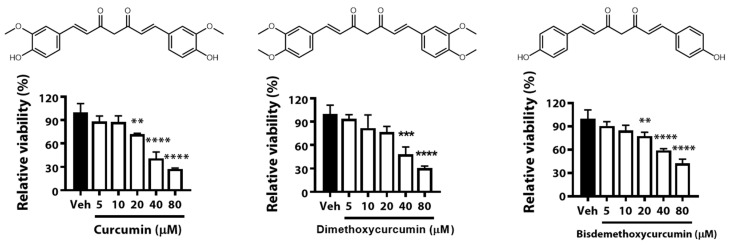
Molecular structures and cytotoxicity of curcumin (CUR), dimethoxycurcumin (DMC), and bisdemethoxycurcumin (BDMC). Viability of B16F10 cells with the treatment of 0, 5, 10, 20, 40, 80 µM of CUR, DMC, and BDMC. Data are presented as mean ± standard deviation (SD). Significant differences are expressed by the symbols **, ***, and **** as ** *p* < 0.01, *** *p* < 0.001, and **** *p* < 0.0001 compared with control.

To set the dose range of CUR, DCM, and BDCM, we performed a Tetrazolium Assay (MTS assay). The inhibition ratio was calculated and represented as a percent compared with the control (Figure 1). We found that the inhibition ratio of the proliferation of B16F10 cells treated with 10 µM of CUR, DCM, and BDCM was 12.31 (±7.90)%, 18.03 (±16.70)%, and 15.14 (±6.74)%, respectively. We also observed that at concentrations of curcuminoids above 20 μM, the percent inhibition ratios of treated cells were above 20% (28.13, 23.26, and 22.38% for cells treated with 20 μM CUR, DCM, and BDCM, respectively). Considering this result, we set the highest dose of curcuminoids to 10 μM when we evaluated their antimelanogenic effect in B16F10 mouse melanoma cells.

### 3.2. Curcumin and Bisdimethoxycurcumin Reduced Total Melanin Contents in B16F10 Mouse Melanoma Cells

**Figure 2:**
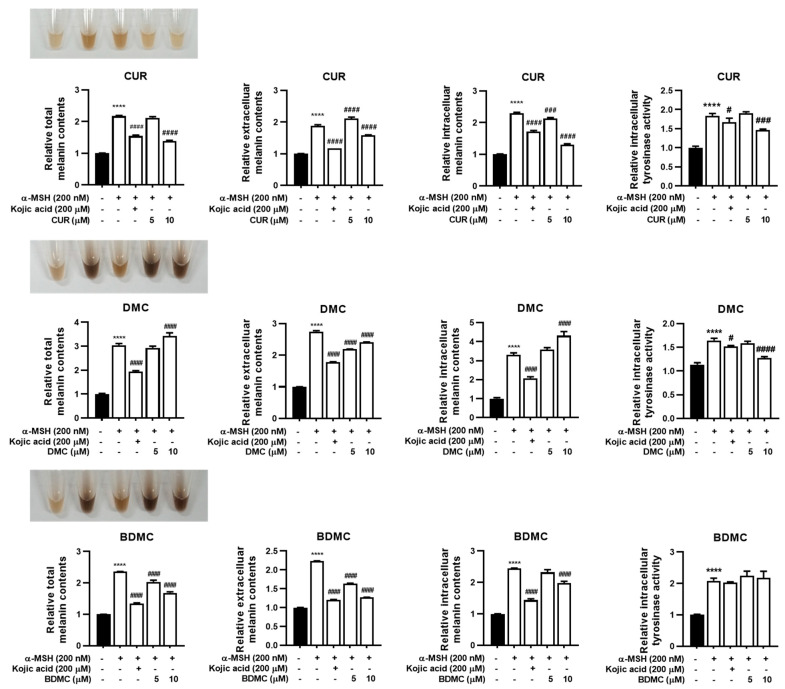
Inhibitory effect of curcumin (CUR), dimethoxycurcumin (DMC), and bisdemethoxycurcumin (BDMC) on alpha-melanocyte stimulating hormone (α-MSH) inducing melanogenesis in B16F10 mouse melanoma cells. The color changes of phenol-free media by accumulation of melanin at 48 h and determined intracellular, extracellular, total melanin contents, and tyrosinase activity after treatment of stimuli (α-MSH), positive control (kojic acid), CUR, DMC, and BDMC, respectively. Data are presented as mean ± standard deviation (SD). Significant differences are expressed by the symbols ****, #, ###, and #### as **** *p* < 0.0001 compared with control and # *p* < 0.05, ### *p* < 0.001, and #### *p* < 0.0001 compared with the α-MSH-treated group, respectively.

We detected that both the extracellular and intracellular melanin contents were elevated in α-MSH-induced B16F10 cells, exhibiting a 1.88- to 2.75-fold and 2.23- to 3.29-fold increase for extracellular and intracellular melanin content, respectively, compared with those in the control (Figure 2). Overall, treatment with α-MSH induced a 2.17- to 3.04-fold increase in total melanin contents in B16F10 cells (Figure 2). We also observed that the stimulation of melanin production in B16F10 cells was suppressed by 1.39- and 1.98-fold after the addition of 5 μM CUR or BDMC, respectively, in comparison to that in the control (Figure 2). We further noticed that at its highest concentration (10 μM), CUR reduced both the extracellular and intracellular melanin contents by 1.59- and 1.31-fold in comparison to those in the control (Figure 2). Likewise, the amount of extracellular and intracellular melanin was 1.69- and 1.28-fold higher after the administration of 10 μM BDMC than that in the control (Figure 2). In contrast, we observed that the addition of 5 and 10 μM DMC increased the production of total melanin in a dose-dependent manner, resulting in 2.94- and 3.43-fold higher total melanin contents, respectively, than those in the control. Moreover, we found that both 10 μM CUR and DMC inhibited the α-MSH-induced increase in tyrosinase activity, keeping it to 1.46- and 1.27-fold higher than that in the control (Figure 2).

### 3.3. Curcumin Derivatives Downregulated the mRNA Levels of Melanin-Production-Related Genes

**Figure 3:**
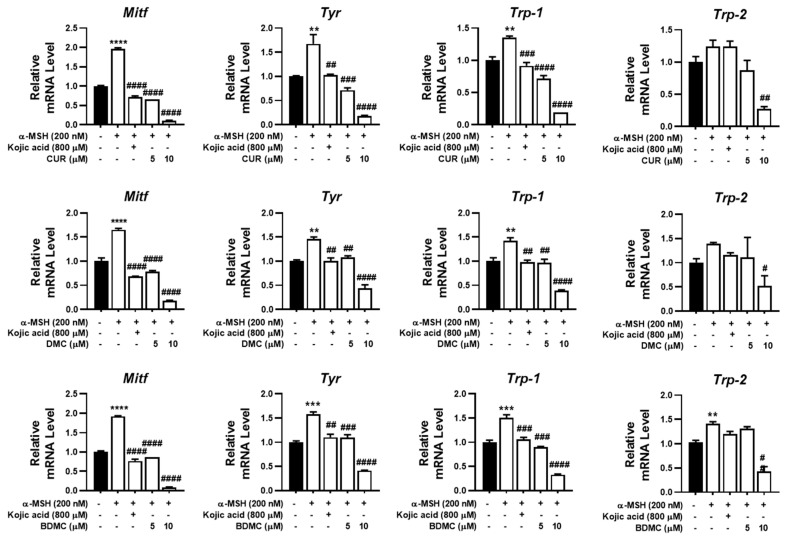
Effect of curcumin, dimethoxycurcumin, and bisdemethoxycurcumin on mRNA level of melanin-biosynthesis-related genes in B16F10 cell line using RT-qPCR. α-MSH, α-melanocyte stimulating hormone; *Mitf*, microphthalmia-associated transcription factor; *Tyr*, tyrosinase; *Trp-1*, tyrosinase-related protein 1; *Trp-2*, tyrosinase-related protein 2. Data are shown as means ± standard deviations (SDs). Significant differences are expressed by the symbols **, ***, ****, #, ##, ###, and #### as ** *p* < 0.01, *** *p* < 0.001, and *** *p* < 0.0001 compared with control, respectively. # *p* < 0.05, ## *p* < 0.01, ### *p* < 0.001, and #### *p* < 0.0001 compared with the α-MSH-treated group, respectively.

We subsequently evaluated the antimelanogenic properties of CUR, DMC, and BDMC at the molecular level. We detected that the α-MSH-induced increases in the mRNA levels of *Mitf*, *Tyr*, *Trp-1*, and *Trp-2* in B16F10 cells were dramatically reduced following treatment with CUR, DMC, or BDMC (Figure 3). We specifically found that the α-MSH-induced increase in the level of *Mitf* mRNA was reduced 0.10-, 0.19-, and 0.08-fold following administration of 10 µM CUR, DMC, or BDMC, respectively, compared with that in the control (Figure 3). Likewise, we observed that the expression of *Tyr* and *Trp-1* was reduced in B16F10 cells following administration of CUR, DMC, or BDMC (5 and 10 µM) in a dose-dependent manner (Figure 3). In contrast, the expression of *Trp-2* was unaltered or slightly reduced following treatment with a low or high dose of curcuminoids, respectively (Figure 3). In particular, the expression of *Trp-2* was reduced by 0.28-, 0.53-, and 0.43-fold following 10 µM CUR, DMC, or BDMC administration, respectively, in comparison to that in the control (Figure 3).

### 3.4. Toxicity of Curcumin Derivatives in Early-Stage Zebrafish Embryos

**Figure 4:**
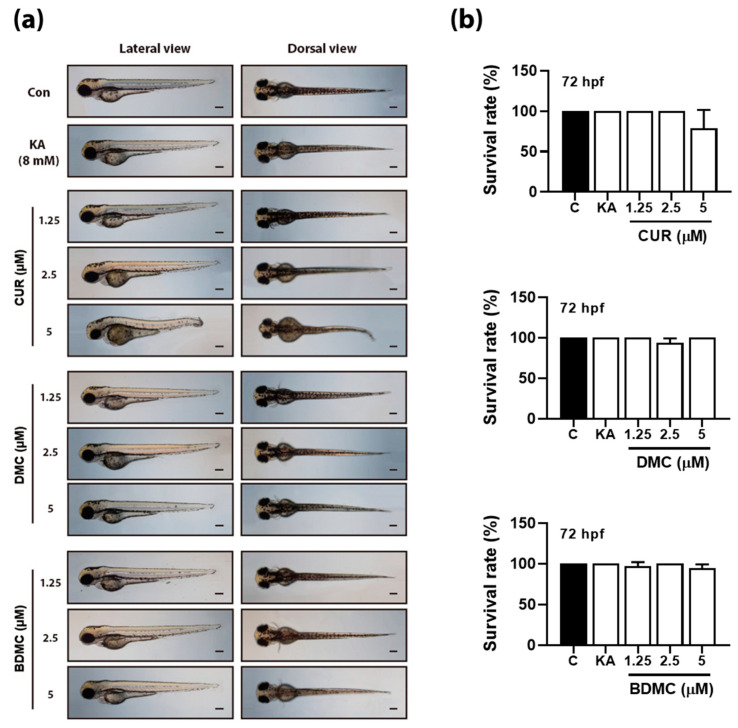
Acute toxicity of curcumin, dimethoxycurcumin and bisdemethoxycurcumin on early-stage development of zebrafish embryo. (**a**) Images of lateral and dorsal view of zebrafish embryo at 72 h after exposure to 8 mM of kojic acid (KA), various concentrations of curcumin (CUR), dimethoxycurcumin (DMC), and bisdemethoxycurcumin (BDMC). (**b**) Survival rate of zebrafish embryos after 72 h of treatment of 8 mM kojic acid or CUR, DMC, and BDMC. Hpf, hours post fertilization.

Before evaluating the antimelanogenic effect of curcuminoids in early-stage zebrafish embryos, we performed acute toxicity tests to set the range of treatment dose. We accordingly observed various malformations in zebrafish embryos treated with 5 μM CUR. More specifically, we found that the spine was curved, and the tail tip was also curved to one side in embryos at 72 hpf (Figure 4). The survival rate was also slightly decreased at 72 hpf (Figure 4). However, these effects disappeared at concentrations of curcuminoids below 2.5 μM. Interestingly, we observed neither abnormal development nor death in zebrafish treated with DMC or BDMC (Figure 4).

### 3.5. Curcumin Derivatives Reduced Melanin Contents in Zebrafish Embryos 

**Figure 5:**
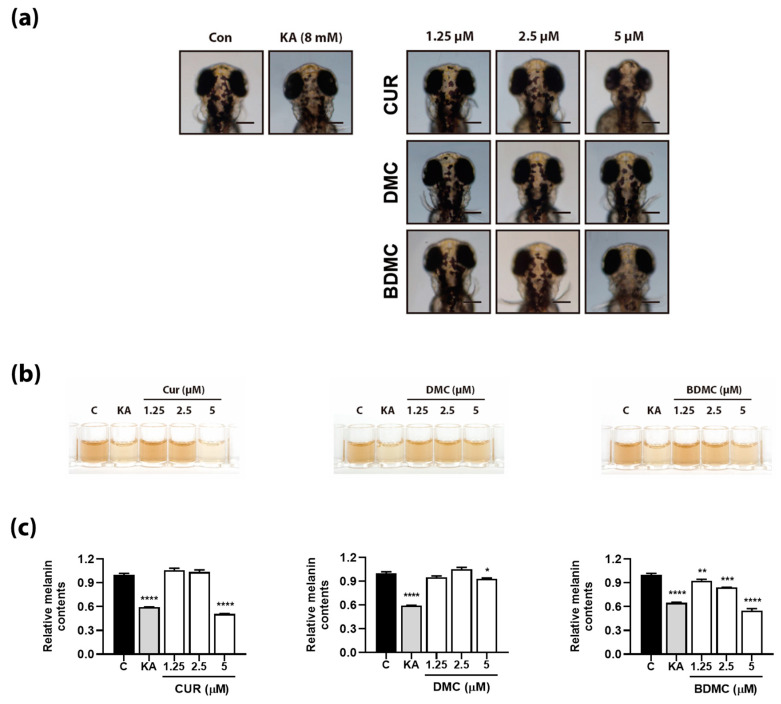
Effect of curcumin (CUR), dimethoxycurcumin (DMC), and bisdemethoxycurcumin (BDMC) on melanin production in zebrafish embryos. (**a**) Images of overhead view of zebrafish embryo at 72 h after exposure of various concentrations of CUR, DMC, and BDMC. (**b**) Color of whole-body lysate with 1 M NaOH. (**c**) Determined absorbance at 490 nm for melanin contents. Data are presented as mean ± standard deviation (SD) of triplicates. Significant differences are expressed by the symbols *, **, ***, and **** as * *p* < 0.05, ** *p* < 0.01, *** *p* < 0.001, and **** *p* < 0.0001, respectively.

We observed the pigmentation of zebrafish as dark dots in the overhead view of zebrafish embryos at 72 h post fertilization (hpf) (Figure 5a). These dots are located between the top of the embryo head and its trunk. Kojic acid, a known antimelanogenic compound, reduces the synthesis of melanin in developing zebrafish embryos, and was thus used here at a concentration of 8 mM as positive control (Figure 5a,b). We determined that kojic acid inhibited the production of melanin by 41% compared with that in the control. We also found that CUR and BDMC inhibited the formation of dark dots on the surface of zebrafish skin in a dose-dependent manner (Figure 5a). More specifically, we detected that administration of 5 µM CUR (highest tested concentration) inhibited the production of melanin in zebrafish by 50% (Figure 5b). Finally, we noticed that although BDMC showed the highest bioactivity in in vitro experiments, it was not the most effective compound in in vivo experiments. In particular, we observed that the relative amount of melanin in zebrafish embryos exposed to 1.25, 2.5, and 5 µM BDMC was 0.92-, 0.84-, and 0.53-fold lower, respectively, than that in the control (Figure 5b).

## 4. Discussion

In this study, we evaluated the potential antimelanogenic effects of CUR, DMC, and BDMC. Among the tested curcuminoids, CUR and BDMC tended to weaken the α-MSH-induced production of melanin. These two compounds reduced not only the intracellular, but also the extracellular melanin contents. Moreover, CUR suppressed the α-MSH-induced increase in tyrosinase activity in B16F10 cells [7]. Notably, the inhibitory effect of CUR was stronger than that of another well-known whitening agent, kojic acid, which has already been reported in many previous studies [20,21,22]. Although the pharmacokinetics study of curcuminoids reported poor bioavailability of these chemicals [23], skin-whitening reagents were not designed for oral administration, but topical cosmetics. In order to overcome this limitation, researchers tried to use curcumin in encapsulated form, and this advanced formulation was very promising [24]. In a previous study, 10 μM CUR was reported to suppress tyrosinase activity by 47.85% [25]. This study’s reported suppression ratio was slightly lower than that in our study; however, their experimental standard deviation was greater. Despite small differences in the inhibition ratio, other studies have also reported the inhibitory effect of CUR against tyrosinase activity and melanin production in B16F10 cells [20,21] and human melanocytes [22,26]. Based on these studies, CUR has strong antimelanogenic activity and can thus be considered a strong candidate whitening agent. In contrast to its antityrosinase activity [27], the antimelanogenic effect of BDMC has not been reported previously. Nonetheless, our in vitro results suggested that BDMC has great potential as a skin-whitening agent. Our quantitative analysis of the mRNA levels of melanin-production-related genes in B16F10 cells revealed that the α-MSH-induced upregulation in the expression of these genes was ameliorated following administration of these three curcumin derivatives. CUR-induced reduction in the level of key proteins in melanogenesis, including MITF, tyrosinase, TRP-1, and TRP-2 was previously reported [21], but changes in the levels of mRNA were not reported. As reported in a previous study, CUR downregulated the expression of members of the MITF-tyrosinase signaling pathway at the protein level [21]. Likewise, our study suggested that the mRNA levels of melanogenesis-related genes were also reduced following treatment with CUR. Combining these findings and those of previous studies, we concluded that CUR inhibits melanogenesis by suppressing the expression of melanogenesis-related genes regulated by the MC1R signaling pathway, including MITF, TYR, TRP-1, and TRP-2 [7,28,29]. Administration of DMC downregulated the expression of melanogenesis-related genes; however, the degree of inhibition was lower than that of CUR and BDMC. In addition, DMC did not lead to a reduction in the total melanin contents in B16F10 cells. To explain these results, further studies are needed. Similar to CUR, BDMC also ameliorated the α-MSH-induced activation of the melanogenesis signaling pathway. To date, BDMC was only known to inhibit tyrosinase [27], which is obviously an enzyme that plays a very important role in the synthesis of melanin; however, melanogenesis is much more tightly regulated by the melanogenesis signaling pathway [7,29]. Hence, we approached it here from a diverse perspective and found that BDMC reduced not only the production of melanin in B16F10 melanoma cells, but also the mRNA level of melanin-production-regulating genes including *Mitf*, *Tyr*, *Trp-1*, and *Trp-2*. This finding suggested that administration of CUR or BDMC ameliorated the MC1R signaling pathway-induced upregulated expression of melanin-production-related genes [7].

In vivo evaluation of the biological activity of a natural compound is one of the most important steps in exploring the potential medicinal use of natural products, because sometimes the biological activity of a compound evaluated in an in vitro system might be different from that exhibited in an in vivo system [18,30]. In addition, the rapid exploration of new candidates is important in the screening phase to reduce time and cost. Therefore, many research groups have been trying to develop faster and more effective in vivo evaluation systems [3,19]. Many studies, including our own, have shown the effectiveness of the zebrafish embryo model for screening compounds with inhibitory activity against melanogenesis [3,17,31]. The main biosynthetic pathways in zebrafish are very similar to those in humans [3]. In addition, the targets of well-known inhibitors of melanogenesis were shown to be similar, and their efficacy has already been confirmed in both the zebrafish embryo model and humans [32,33]. In this study, we screened three curcumin derivatives for their antimelanogenic properties using zebrafish embryos. None of the tested curcuminoids showed acute toxicity at concentrations below 5 μM in 96 hpf zebrafish. However, at a concentration of 5 μM, both abnormal development of zebrafish embryos and lethality were confirmed. Notably, 5 μM CUR was shown to result in the tail bending of zebrafish embryos. A previous study also reported the malformation of zebrafish embryos in the presence of CUR [34]. In that study, the toxic effects of CUR were reported to occur in a dose-dependent manner [34], with a minimum effective concentration of 5 μM, which is consistent with our findings [34]. Considering the malformations observed in zebrafish embryos, we concluded that CUR is cytotoxic in zebrafish embryos during early development.

Both CUR and BDMC exhibited outstanding inhibition properties in the in vivo test. Importantly, the inhibitory effect of both curcuminoids was stronger than that of kojic acid, a compound used as positive control in our study and one of the most popular whitening agents in the cosmetic industry [6]. In addition, the concentration of kojic acid was 8 mM, which was much higher than that of CUR (5 µM) or BDMC (5 µM). Another compound frequently used as positive control in antimelanogenic studies, arbutin, has also been used at a concentration range of 10 to 20 mM [3,35,36], with both compounds, arbutin and kojic acid, being reported to have similar activity and working concentrations [3]. Compared with these positive control compounds, the working concentration of our two candidate agents was much lower (5 µM for curcuminoids vs. 10 mM for the two positive controls) in the in vivo investigation of melanin production. Hence, these findings suggested that our curcuminoid candidate compounds have great potential to be used as whitening agents in cosmetics.

## 5. Conclusions

In conclusion, as all these results demonstrate, CUR and BDMC have outstanding inhibition effects on melanin production. Considering the toxic effect of CUR on zebrafish embryos during early-stage development, BDMC may be used as a whitening agent for melanogenesis by inhibiting melanin-production-related proteins TYR, TRP-1, and TRP-2 in both in vitro and in vivo. 

## Data Availability

The data presented in this study are available on request from the corresponding author. The data are not publicly available due to privacy or ethical restrictions.

## Supplementary Materials

The following supporting information can be downloaded at: https://www.mdpi.com/article/10.3390/foods12050926/s1, Table S1: Primer list.
