# Supplementary material for: Antimelanogenic Effects of Curcumin and Its Dimethoxy Derivatives: Mechanistic Investigation Using B16F10 Melanoma Cells and Zebrafish (Danio rerio) Embryos
Source: Foods. 2023 Feb 22;12(5):926. doi: 10.3390/foods12050926 (PMC10000565; doi:10.3390/foods12050926)
Supplement: Supplementary file 1 [file foods-12-00926-s001.zip › foods-2194761-supplementary.pdf]

Table S1. Primer list used for RT-qPCR.

Gene symbol	Gene name	Primer sequence (5′-3′)
Mitf	Melanogenesis associated transcription factor	F) CATCATCAGCCTGGAATCAA<br>R) TCAAGTTTCCAGAGACGGGT
Tyr	Tyrosinase	F) TCTTCACCATGCTTTTGTGG<br>R) ATAGGTGCATTGGCTTCTGG
Trp-1	Tyrosinase-related protein 1	F) TGGTCTGTGAATCCTTGGAA<br>R) CATTTCCAGCTGGGTTTCTC
Trp-2	Tyrosinase-related protein 2	F) CGTGCTGAACAAGGAATGC<br>R) CGAAGGATATAAGGGCCACTC
Gapdh	Glyceraldehyde-3-phosphate dehydrogenase	F) GGTTGTCTCCTGCGACTTCA<br>R) TGGTCCAGGGTTTCTTACTCC
